# Longitudinal analysis of vitamin D levels considering sunshine duration and suggestion for a standardised approach for vitamin D supplementation in children and adolescents with obesity

**DOI:** 10.1186/s12887-024-04823-x

**Published:** 2024-05-15

**Authors:** Katja Wechsung, Dirk Schnabel, Susanna Wiegand

**Affiliations:** Department for Pediatric Endocrinology and Diabetology, Center for Chronically Sick Children, Charité - Universitätsmedizin Berlin, Corporate Member of Freie Universität Berlin, Humboldt-Universität zu Berlin, Berlin Institute of Health, Augustenburger Platz 1, 13353 Berlin, Germany

**Keywords:** Obesity, Children, Adolescents, Vitamin D, Sunshine duration, Rickets

## Abstract

**Background:**

Children with obesity have low 25 hydroxy-vitamin D (25-OH-D_3_) levels compared to lean children. Recommendations on when to start vitamin D supplementation differ largely between countries. Longitudinal data on 25-OH-D_3_ levels to guide treatment decisions are scarce since they are largely influenced by solar radiation and are difficult to compare.

**Methods:**

We carried out a retrospective analysis of multiple 25-OH-D_3_ and parathyroid hormone (PTH) measurements in a cohort of 543 patients without vitamin D supplementation. All measurements were taken at the local paediatric obesity clinic as documented in the German-Austrian-Swiss APV (Prospective Documentation of Overweight Children and Adolescents) registry from 2009 to 2019. Serial 25-OH-D_3_ and PTH levels were adjusted for sunshine duration over the last 30 days to account for seasonal variation, as well as for sex and body mass index (BMI). We further performed an exploratory analysis of the association of sunshine duration, sex, BMI SDS (standard deviation score), abnormal lipid levels or dysglycemia with the 25-OH-D_3_ trend.

**Results:**

229 obese patients (mean BMI SDS: 2,58 (± 0,56), 53% females, mean age: 12 (± 3) years, range: 2–21 years) with two, 115 with three and 96 with four repeated 25-OH-D_3_ measurements were identified. Mean adjusted 25-OH-D_3_ (48.2 nmol/l) and PTH (34.9 ng/l) levels remained stable over 120 weeks. 5% of the patients had an elevated PTH > 65 ng/l. High total cholesterol ≥ 200 mg/dl and high triglycerides ≥ 130 mg/dl were associated with higher 25-OH-D_3_ levels.

**Conclusion:**

We propose a simple method to include sunshine duration in the analysis of 25-OH-D_3_ levels to minimise the bias of seasonal variation. Based on our data we established the pragmatic strategy of limiting vitamin D supplementation to patients with biochemical signs of mineralisation disorders such as elevated PTH and alkaline phosphatase (AP). In children with normal PTH and AP we recommend adjustment of calcium intake and increase of outdoor activity instead.

## Background

Vitamin D is essential for bone health in the growing skeleton. Vitamin D deficiency and/or low calcium intake can lead to nutritional rickets with defective mineralisation [1]. Moreover, vitamin D may play a role in the development of mental, immunological or cardiometabolic diseases [2]. The standard laboratory parameter for vitamin D supply is 25 hydroxyvitamin D (25-OH-D_3_) [3].

Numerous cross-sectional studies have documented lower 25-OH-D_3_ levels in children with obesity compared to normal weight control groups and hypothesised a negative effect on cardio-metabolic parameters [4, 5]. Although the bioavailability of total 25-OH-D_3_ is reduced, free 25-OH-D_3_ seems to be comparable in lean and overweight children [6, 7]. Levels of 25-OH-D_3_ normalise with weight loss [8]. This supports the hypothesis that body composition and vitamin D storage in fat tissue are the main reasons for low 25-OH-D_3_ levels in patients with obesity [9]. Large randomised controlled intervention studies in adults and smaller studies in children have shown no effect of vitamin D supplementation on weight, cardiovascular risk factors, or metabolic parameters [10–13]. In children with 25-OH-D_3_ levels above 50 nmol/l, vitamin supplementation does not improve bone health [14–16].

Consequently, the current standard practice in the care of normal-weight children in Germany is to refrain from vitamin D testing and supplementation beyond infancy [17].

However, the long-term effects of persistently low 25-OH-D_3_ levels on the bone health of children with obesity are still largely unknown, and guidelines differ on the recommendations for optimum 25-OH-D_3_ serum levels and vitamin D supplementation [18]. A cross-sectional study found an increased fracture risk to be associated with 25-OH-D_3_ levels below 50 nmol/l, which might justify vitamin D supplementation [19]. However, even in these cases, supplementing obese children with vitamin D might not always be advisable. Other studies suggest that it leads to increased cholesterol and triglyceride levels in adolescents without changing their BMI [20], warranting special caution as to possible adverse effects of vitamin D supplementation on metabolic comorbidities in patients with obesity. This leaves open questions for the paediatrician regarding when, and for whom, to start vitamin D supplementation in routine care for children with obesity.

For the outpatient program of the paediatric obesity clinic at Charité - Universitätsmedizin Berlin, we aimed to establish a pragmatic approach focusing narrowly on the effect of vitamin D on bone mineralisation. Calcipenic disorder of bone mineralisation is usually detected by low levels of 25-OH-D_3_ in conjunction with elevated levels of parathyroid hormone (PTH) above 65 ng/l (normal range: 15,0–65,0 ng/l) and elevated alkaline phosphatase (AP) [21]. The goal of the present study is to evaluate follow-up data of unsupplemented children with obesity. We had the question about whether PTH levels remain in the normal range over time in children without vitamin D supplementation or whether a negative trend in 25-OH-D_3_ and elevation PTH were apparent, suggesting a structurally deficient endogenous vitamin D production and prompting vitamin D supplementation to minimise the risk for development of rickets or osteomalacia.

The longitudinal analysis of vitamin D levels is considerably complicated by the presence of seasonal effects. Cutaneous synthesis induced by solar UV-B radiation of 290–315 nm is the principal source of vitamin D for humans [22]. Geographical and seasonal changes in UV radiation lead to a physiological variability of serum 25-OH-D_3_ levels [23]. This seasonal (and geographical) effect on observed 25-OH-D_3_ levels is a major confounder in vitamin D studies [24]. A recent analysis demonstrates that even at the same date and location, sunshine duration, and therewith endogenous vitamin D production, are highly variable between years [25]. 25-OH-D_3_ levels are particularly volatile in spring and autumn. As a novel approach, we use meteorological data on the duration of sunshine at the time of vitamin D measurements to account for seasonal confounding. This contrasts with the standard approach to merely account for the month the measurements were taken that ignores differences present between years.

Our study adds to the scarce long-term follow-up data of 25-OH-D_3_ levels in combination with PTH in children with obesity. To our knowledge, it is also the first study of children to incorporate actual sunshine duration to account for seasonal variation. In so doing, we hope to contribute to establishing an informed standard of care regarding vitamin D supplementation for children with obesity.

## Methods

### Patients

The program of the outpatient paediatric obesity clinic at Charité-Universitätsmedizin Berlin offers medical care and structured counselling on physical exercise and nutrition by a multidisciplinary team. After the patients´ and guardians´ consent, data from the clinic chart are anonymised according to data management guidelines and routinely entered in the German / Swiss / Austrian obesity registry APV.

The APV registry is a standardised multicentre database for the prospective documentation of anthropometric, demographic and metabolic parameters in overweight or obese children. Anonymised data are aggregated into a cumulative database for clinical research and quality assurance, as described previously [26, 27]. The APV initiative is authorised by the Ethics Committee of the University of Ulm, Germany and approved by the local review board of Charité Universitätsmedizin Berlin.

For the present analysis data from the local paediatric obesity clinic were used. We identified 543 paediatric patients aged 0–18 years that participated in the outpatient program of the paediatric obesity clinic, Charité Universitätsmedizin Berlin, from 2009 to 2019 and had documented 25-OH-D_3_ and PTH levels, age, sex, medication, parent migration background (born outside Germany, Austria, or Switzerland yes/no), Body mass index standard deviation score (BMI SDS) and metabolic parameters (LDL cholesterol, HDL cholesterol, total cholesterol, triglyceride, fasting glucose and insulin) in the APV database. Up to 4 consecutive measurements of 25-OH-D_3_ and PTH were identified for these patients and included in the analysis. The intervals between blood collections were not predefined as inclusion criteria but calculated as means from the data retrospectively. Hence, the distance between time points differed between the patients. We excluded 637 patients with missing values and 14 patients with documented oral vitamin D intake. BMI SDS for age and sex was calculated from height (obtained by wall-mounted stadiometer nearest to 0,1 cm) and weight (obtained by standardised scale nearest to 0,1 kg) using German national representative reference values [28]. Obesity was defined as a BMI above the 97th percentile according to German guidelines [29].

### Blood testing

Blood was drawn in a fasting state in the morning. Reasons for repeated blood measurements were the follow-up on metabolic comorbidities such as dysglycemia or hyperlipidemia or low 25-OH-D_3_ values (reference range 50–150 nmol/l). 25-OH-D_3_ was determined by a chemiluminescence immunoassay (IDS total 25-OH Vitamin D, IS-2500 N, Immunodiagnostic systems, United Kingdom). Intact PTH (reference range 15–65 ng/l) was determined by an electro-chemiluminescence immunoassay (ElecsysT PTH (1–84), Roche Diagnostics, Mannheim, Germany). LDL, HDL cholesterol, total cholesterol and triglycerides were measured with a homogeneous enzymatic colourimetric assay by the automated Roche/Hitachi cobas c Systems (HDL cholesterol plus 3rd generation, LDL cholesterol plus 2nd generation, Cholesterol Gen. 2, Triglyceride, Roche Diagnostics, Mannheim, Germany). Glucose was determined with a homogeneous enzymatic colourimetric assay by the automated Roche/Hitachi cobas c Systems (Glucose HK Gen.3, Roche Diagnostics, Mannheim, Germany) and insulin was determined with electro chemiluminescence immunoassay (Elecsys Insulin, Roche Diagnostics, Mannheim, Germany).

Lipid profile was categorised as follows: high total cholesterol ≥ 200 mg/dl; high low-density lipoprotein cholesterol (LDL) ≥ 130 mg/dl; low high-density lipoprotein cholesterol (HDL) < 35 mg/dl; high triglycerides ≥ 130 mg/dl. Dysglycemia was assessed by fasting glucose. Impaired fasting glycemia (IFG) was defined as a fasting glucose 100–125 mg/dl. Elevated Insulin was defined as ≥ 14 mU/l.

### Sunshine duration

Information about the daily sunshine duration (in hours) in Berlin was obtained from the records of the German weather service (Deutscher Wetterdienst). The raw data were interpolated for Berlin, Germany (longitude/latitude coordinates 13.444/52.556) to fill in for missing values at single weather stations.

The half-life of 25-OH-D_3_ is estimated to lay between 14 and 27 days [30, 31]. We calculated a mean of the daily sunshine duration over the last 30 days (Sun 30) before the date of the blood sampling.

### Statistical analysis

Statistical analysis was performed using SPSS IBM Statistics (version 28.0) and SAS (version 9.4). As descriptive measures, we calculate relative frequencies for categorical variables and mean with standard deviation for continuous variables. We use linear regression models (ANCOVA) with 25-OH-D_3_ and PTH as dependent variables, respectively, and the four time points (T1, T2, T3, T4). All models were adjusted for sex (categorical), BMI SDS, and Sun 30 (continuous) as explanatory variables. For the model with 25-OH-D_3_ as the dependent variable, we include PTH as an explanatory variable as well and vice versa. We report adjusted means with 95% confidence intervals. We looked at the potential association of abnormal metabolic parameters (dysglycemia, fasting insulin and abnormal lipid profile) and 25-OH-D_3_ levels. Since all analyses are of an explorative nature, we do not adjust for multiple testing. Therefore all p-values should be considered descriptive.

## Results

We included 543 patients with one 25-OH-D_3_ measurement. Of these 543 patients, 229 patients had two, 115 patients had three, and 96 patients had four serial 25-OH-D_3_ measurements. Table 1 gives details of the patients with up to four repeated 25-OH-D_3_ measurements.

**Table 1:** Characteristics of patients with four measurements of 25-OH-D3.

Time point	1	2	3	4
Number of patients	543	229	115	96
Time to next measurement (weeks)	40,7 (± 33,5)	47,7 (± 20,11)	31,9 (± 16,79)	-
Mean age (years)	12 (± 3)	13 (± 3)	15 (± 2)	15 (± 2)
Age range (years)	2–21	3–19	11–19	11–20
Mean BMI SDS	2,58 (± 0,56)	2,4 (± 0,64)	2,27 (± 0,58)	2,25 (± 0,57)
BMI SDS range	0,66 − 4,67	0,92 − 4,28	0,39 − 3,6	0,11 − 3,43
Sex female	288 (53,0%)	117 (51,1%)	57 (49,6%)	49 (51,0%)
Sex male	255 (47,0%)	112 (48,9%)	58 (50,4%)	47 (49,0%)
Migration Background Mother	291 (54%)	82 (34%)	40 (35%)	35 (36%)
Migration Background Father	330 (61%)	129 (56%)	56 (49%)	47 (49%)
Unadjusted 25-OH-D3	47,5 (10–127)	48,2 (8-116)	48,5 (12–253)	43 (12–116)
Patients with 25-OH-D3 < 50 nmol/l	323 (59%)	130 (57%)	68 (59%)	70 (72%)
Patients with 25-OH-D3 < 30 nmol/l	101 (19%)	47 (20%)	20 (17%)	33 (34%)
Patients with PTH > 65 ng/l	27 (5%)	10 (4%)	0	3 (3%)
Sun 30 (hours)	4,47 (± 2,64)	5,19 (± 2,83)	5,48 (± 2,45 )	4,03 (± 2,50)
Mean Triglycerides (mg/dl)	103 (± 52)	101 (± 55)	90 (± 45)	87 (± 41)
Mean Total cholesterol (mg/dl)	161 (± 31)	154 (± 31)	161 (± 32)	157 (± 31)
Mean HDL (mg/dl)	48 (± 11)	47 (± 10)	49 (± 11)	50 (± 11)
Mean LDL (mg/dl)	101 (± 29)	96 (± 29)	97 (± 30)	93 (± 30)
Mean Fasting glucose (mg/dl)	82 (± 8)	82 (± 9)	82 (± 9)	81 (± 7)
Mean Fasting insulin (mU/l)	22 (± 21)	22 (± 21)	18 (± 13)	17 (± 11)
Patients with high triglycerides	124 (23%)	53 (24%)	18 (16%)	14 (15%)
Patients with high total cholesterol	62 (11%)	19 (8%)	14 (12%)	8 (8%)
Patients with low HDL	51 (9%)	26 (12%)	9 (8%)	4 (4%)
Patients with high LDL	77 (14%)	25 (11%)	15 (13%)	9 (9%)
Patients with impaired fasting glucose	0	1 (0,4%)	1 (0,9%)	0
Patients with elevated fasting insulin	354 (65%)	136 (60%)	55 (49%)	47 (49%)

Data are presented as mean ± SD, range or number of patients (percentages). BMI, body mass index; HDL, high-density lipoprotein; LDL, low-density lipoprotein; PTH, intact parathyroid hormone; SDS, standard deviation score; Sun 30, Mean sunshine duration over the last 30 days; 25-OH- D_3_: 25 hydroxy vitamin D.

Metabolic parameters were categorised as follows: high total cholesterol ≥ 200 mg/dl; high low-density lipoprotein cholesterol (LDL) ≥ 130 mg/dl; low high-density lipoprotein cholesterol (HDL) < 35 mg/dl; high triglycerides ≥ 130 mg/dl. Impaired fasting glycemia 100–125 mg/dl, elevated fasting insulin ≥ 14 mU/l.

The interval between the first and the last measurement adds up to 120 weeks (2 ½ years). The mean age of the patients lies in the adolescent range (12 ± 3 years). Males and females are evenly distributed in the group. The mean BMI SDS and percentage of children with migration backgrounds decreased from the first to the fourth measurement of 25-OH-D_3_.

**Fig. 1:**
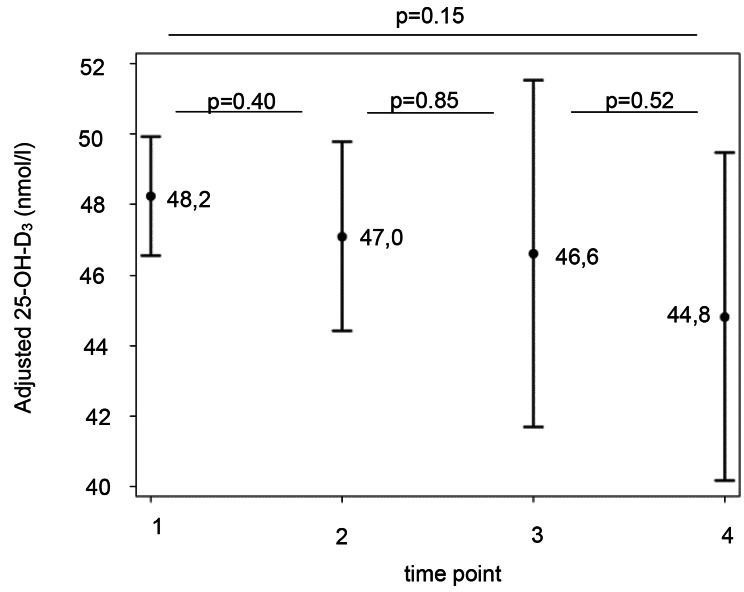
Mean adjusted 25-OH-D_3_ levels at the four time points. 25-OH-D_3_ levels and 95% confidence intervals adjusted for sex, BMI SDS and Sun 30 and. P-values were generated using ANCOVA. BMI, body mass index; SDS, standard deviation score; Sun 30, Mean sunshine duration over the last 30 days; 25-OH- D_3_, 25 hydroxy vitamin D

**Fig. 2:**
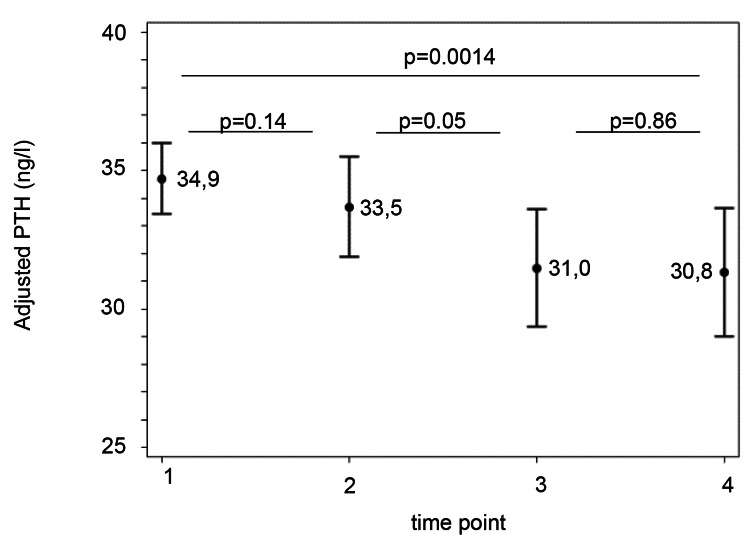
Mean adjusted PTH level at four time points. Mean PTH levels and 95% confidence intervals adjusted for BMI SDS, Sun 30 and 25-OH-D3 levels. P-values were generated using ANCOVA. BMI, body mass index; PTH, parathyroid hormone; SDS, standard deviation score; Sun 30, Mean sunshine duration over the last 30 days; 25-OH- D_3_, 25 hydroxy vitamin D

At time T1, more than 50% of the patients, showed an unadjusted 25-OH-D_3_ level below 50 nmol/l, and 20% showed levels below 30 nmol/l. These proportions increased to 66% and 33%, respectively, at the fourth measurement (T4). By contrast, the 25-OH-D_3_ values adjusted for sun 30, BMI SDS, and sex remained stable over time (Fig. 1). The percentage of children with elevated PTH above the cut-off of 65 ng/dl did not rise between T1 and T4. The longitudinal PTH levels adjusted for 25-OH-D_3_, sun 30, and BMI SDS remained stable over 2 ½ years (Fig. 2).

Influencing factors on 25-OH-D_3_ levels are depicted in Table 2. We found a positive association of Sun 30, high triglycerides, high LDL and high total cholesterol with 25-OH-D_3_ levels, respectively. Male sex and impaired fasting glucose had a negative association.

Sun 30 (ß = − 0.43; *p* = 0.03) and 25-OH-D_3_ level (ß= − 0.26; *p* < 0.01) had a negative association with PTH levels.

**Table 2:** Influence of patient characteristics, sun 30 and abnormal metabolic parameters on 25-OH-D_3_ levels

	ß	*p*
BMI SDS	− 0.02	0.98
Male sex	-2.28	0.18
Sun 30	2.48	< 0.001
High triglycerides	10.58	0.015
High total cholesterol	6.58	0.031
Low HDL	-0.18	0.95
High LDL	8.78	0.09
Impaired fasting glucose	-12.95	0.05
Elevated fasting insulin	1.22	0.45

Estimated ß coefficients with respective p-value generated with ANCOVA

BMI, body mass index; SDS, standard deviation score; Sun 30, mean sunshine duration over the last 30 days; 25-OH- D_3_, 25 hydroxy vitamin D

Metabolic parameters were categorised as follows: high total cholesterol ≥ 200 mg/dl; high low-density lipoprotein cholesterol (LDL) ≥ 130 mg/dl; low high-density lipoprotein cholesterol (HDL) < 35 mg/dl; high triglycerides ≥ 130 mg/dl. Impaired fasting glycemia 100–125 mg/dl, elevated fasting insulin ≥ 14 mU/l.

## Discussion

In this study, we retrospectively evaluated longitudinal 25-OH-D_3_ levels in a large cohort of children with obesity without vitamin D supplementation. We use a novel method to adjust 25-OH-D_3_ data for sunshine duration. We show that the mean adjusted 25-OH-D_3_ and PTH levels remain stable over 120 weeks (2 ½ years).

Based on this data we established the following standard of care for children with overweight in our obesity clinic:

- Measurement of 25-oh-D levels only in combination with PTH, AP, serum calcium and phosphate.
- No vitamin D supplementation for low 25-OH-D3 levels if PTH and AP are normal. Instead, we advice regarding adequate calcium intake and recommend regular outdoor activity.

We prescribe vitamin D supplementation only if PTH is increased above 65 ng/dl (normal range: 15,0–65,0 ng/l) and AP is elevated. For low 25-OH-D_3_ levels without elevated PTH or AP, we will recommend regular outdoor activity aiming to increase endogenous vitamin D production through sun exposure and to promote physical exercise as an important stimulus for bone remodelling [32]. These recommendations may have an additional positive impact on weight development and avoid the drawbacks of liberal vitamin D supplementation [33–35]. Patients at special risk of vitamin D deficiency e.g. with dark or largely covered skin will receive an individualised follow-up on 25-OH-D_3_ levels.

According to this approach 5% of our cohort with elevated PTH required treatment for biochemical signs of osteomalacia. In this study we used the upper limit of normal of 65 ng/l for PTH. Values above this cut off are observed in rickets and osteomalacia [36, 37]. Thresholds such as 50ng/l or 45 ng/l have been proposed for PTH in children [38–40]. Low calcium intake elevates PTH and complicates the quest for a PTH threshold. Two studies analysed longitudinal 25-OH-D_3_ and PTH levels in 32 German children [8] and 67 Spanish children [41] with obesity over 12 months. In both studies, 25-OH-D_3_ levels remained stable, which is consistent with our findings. In the Spanish cohort, the number of children with 25-OH-D_3_ levels below 20 ng/ml (50 nmol/l) increased from 32 to 41% over 12 months. Though the mean 25-OH-D_3_ levels were higher in the Spanish group, the children showed higher mean PTH levels compared to our data [42]. The authors found no seasonal variance in PTH compared to lean children and argued that obesity might be a confounding factor. A causative relation between low 25-OH-D_3_ and elevation of PTH in patients with obesity is still a matter of discussion. Several studies show that PTH suppression thresholds vary with body weight and may not reflect the actual vitamin D status [43]. From a pathophysiological view, raised PTH and low 25-OH-D_3_ levels indicate the need for additional calcium or vitamin D intake to improve bone mineralisation. Our data show an inverse association of 25-OH-D_3_ and PTH, accordingly. Longitudinal data in children with obesity with low 25-OH-D_3_ and documented calcium intake are necessary to define PTH thresholds that require treatment with vitamin D and/or calcium.

Values for AP, serum calcium and phosphate were not available for analysis in our patients. There was no difference in AP, serum calcium and phosphate in the group of German children with obesity after 12 months [8]. Likewise, serum calcium and phosphate remained stable in the Spanish group but AP was not determined [41].

Interestingly, sunshine duration or other parameters of radiation are not included in the evaluation of 25-OH-D3 levels even in recent large association or treatment studies both in adults and children [44]. One recent study used day- and place specific UV radiation data from meteorological satellites to analyze the influence of radiation on 25-OH-D3 levels in a large population of healthy children in Europe. This study found that UV radiation and outdoor time were the most important determinants.

of 25-OH-D3 levels [45].

The existing studies in children with obesity re-evaluated the children after exactly one year to minimise the impact of seasonal variance [8, 41]. In children with obesity, the relevance of a seasonal influence is occasionally questioned as they are suspected to spend little time outdoors and to have reduced sun exposure [42]. Yet, according to our data, even the short periods of time these children spend outside notably influence their 25-OH-D_3_ levels. This is in line with the recommendation that a daily outdoor activity of 15 min between 10:00 am and 3:00 pm is sufficient to promote endogenous vitamin D synthesis from April to September in Germany (geographic location between 48Æ and 54Æ Northern latitude) and may correspond to the way from school or running an errand in the afternoon [46, 47]. Future research should focus on the role and the use of sunshine duration or similar parameters of sun radiation in the interpretation of 25-OH-D_3_ levels in clinical practice.

As to the influence of different metabolic parameters on the 25-OH-D_3_ concentration, fasting insulin and fasting glucose do not seem to have a high explanatory potential. By contrast, elevated triglyceride and total cholesterol levels were strongly associated with higher 25-OH-D_3_ levels. Given the nature of vitamin D as being fat-soluble and its association with lipid metabolism [10], this finding may not be surprising.

Although the effects of vitamin D supplementation on lipid levels in adults and children remain contradictory [5, 13, 48], previous studies suggest that it leads to increased cholesterol and triglyceride levels in adolescents [20]. These results should advise caution to the adverse effects of vitamin D supplementation on metabolic comorbidities in patients with obesity. All the more so as higher vitamin D doses are needed to achieve a normalisation of 25-OH-D_3_ levels in overweight patients and daily and weekly dosing regimens differ [49]. A further drawback of liberal vitamin D supplementation is vitamin D toxicity. Cases of vitamin D intoxication are mainly reported in infants with mis-dosage of soluble vitamin D resulting in severe hypercalcemia, failure to thrive and nephrocalcinosis [34]. Possible harmful effects of chronic moderate vitamin D dosing are discussed [34]. High vitamin D doses might be required for the treatment of osteomalacia or rickets and should be accompanied by monitoring for urinary calcium excretion and nephrocalcinosis [33].

There are several limitations of our study. The data were analysed retrospectively. The statistical analysis involved multiple comparisons and have exploratory character. There is a probability that vitamin D intake was underreported in our patients since vitamin D is an easily available over-the-counter drug. Patients with multiple 25-OH-D_3_ measurements adhered to the multimodal treatment in the obesity clinic. This might explain differences in BMI-SDS and the percentage of children with migration backgrounds between the time points. For patients that dropped out of treatment, no repeated measurements were available. Skin pigmentation and eating habits of different cultures have an impact on 25-OH-D_3_ levels [50]. No data on ethnicity or skin pigmentation was available for analysis. Further data is needed to assess the role of ethnicity in children with obesity. One-third of our patients stem from parents with a migration background. This includes a high proportion of families of Turkish descent [51]. Vacation time in the Mediterranean area could have possibly elevated 25-OH-D_3_ levels in this cohort. Although a cross-sectional analysis of 25-OH-D_3_ levels in Turkish school children suggests that a stay in southern latitudes does not automatically optimise 25-OH-D_3_ levels [52]. Pubertal status, AP, serum calcium and phosphate levels, daily outdoor activity, calcium intake or imaging were not available for analysis in this study. All these parameters could provide more detailed information on the effect of sun radiation and 25-OH-D_3_ levels on bone mineralisation and structure. No longitudinal data exist on the bone mineral density (BMD) of vitamin D-deficient children or adolescents with obesity. Studies examining the effect of 25-OH-D_3_ levels on BMD with Dual-energy X-ray absorptiometry (DXA) or peripheral quantitative CT (pQCT) report contradictory findings. While they do not show an association of 25-OH-D_3_ levels with BMD before puberty a higher level of 25-OH-D_3_ is associated with higher BMD in early adulthood [53]. Further studies that combine all these parameters in a longitudinal analysis are necessary to assess bone health in obese children with low 25-OH-D_3_ levels. Especially the prospective analysis of the group of patients with low 25-OH-D_3_ levels and elevated PTH and AP are important to define risk factors and understand the impact on their bone health. We hope to provide further data in the future.

## Conclusions

We propose including parameters of sun radiation in all future studies concerning vitamin D in children with obesity to avoid the over-interpretation of physiologic fluctuations in 25-OH-D_3_ levels. Sunshine duration in hours is a parameter that is easily obtainable from local weather stations and can be included in the interpretation.

We suggest the pragmatic strategy of limiting vitamin D supplementations to patients with biochemical signs of mineralisation disorders such as elevated PTH and AP. Starting vitamin D supplementation based on a single 25-OH-D3 value without the regard of season, radiation or AP and PTH level might be instead harmful and costly [35]. Instead we educate patients with low 25-OH-D_3_ and normal PTH and AP levels on the necessity of regular outdoor activity and a calcium-rich diet. Further studies on the bone health of obese children with low 25-OH-D_3_ and elevated PTH and AP are needed to evaluate this approach.

## Data Availability

The data that support the findings of this study are available from APV registry but restrictions apply to the availability of these data, which were used under license for the current study, and so are not publicly available. Data are however available from the authors upon reasonable request and with permission of APV registry scientific panel. Please contact Katja Wechsung with data requests.

## Abbreviations

AP: Alkaline phosphatase
APV: Prospective Documentation of Overweight Children and Adolescents
BMI: Body mass index
HDL: High-density lipoprotein
LDL: Low-density lipoprotein
PTH: Intact parathyroid hormone
SDS: Standard deviation score
Sun 30: Mean sunshine duration over the last 30 days
UV-B: Ultraviolet B
25-OH-D3: 25 hydroxy vitamin D
